# The characterization of functional conduction block in patients with multiple types of atrial tachycardia- A discussion on the mechanism of multiple atrial tachycardia

**DOI:** 10.1007/s10840-024-01817-8

**Published:** 2024-05-21

**Authors:** Bin Zhu, GuoHua Zhang, SongCai Xie, Ying Luan, Wei Cao, Jian Xu, Shuo Zhang, JinWei Tian, Fan Wang, ShuFeng Li

**Affiliations:** 1 Department of Cardiology, 2, Affiliated Hospital of Harbin Medical University, Harbin, China; 2 Department of Cardiology, The Second Hospital of Harbin, Harbin, China; 3 Heilongjiang Provincial Key Laboratory of Panvascular Disease, Harbin, China

**Keywords:** Atrial tachycardias, Functional conduction block, Catheter ablation, Reentry, Scar

## Abstract

**Background:**

High-resolution mapping offers superior accuracy in delineating conduction features; however, certain characteristics are still linked to elevated recurrence rates of atrial tachycardia (AT), suggesting the influence of additional mechanisms. This study systematically assessed the substrate of functional conduction block (FCB) regions in relation to the mechanisms of multiple ATs.

**Methods:**

In this study, the Carto system facilitated the mapping of ATs in 13 patients undergoing ablation, each presenting with more than two AT variants. FCB regions were marked and further analyzed.

**Results:**

A total of 33 sustained ATs were mapped across the patient cohort. FCB regions showed convertibility in 7 of 13 patients (54%). Three kinds of presentations can be summarized by the FCB region: Firstly, the FCB region could act as the main obstacle sustaining the localized reentrant pathway, for which rounding obviously has a direct correlation with the mechanism of the AT (27%). Secondly, the FCB regions could act as obstacle lines to reorganize the propagation of the reentry in localized AT and macroreentrant AT (55%). Lastly, the FCB region could act as a bystander and may not be related to the mechanism of the ATs (18%). The potentials in FCB regions mostly performed low voltages or fragmented potentials (FPs) in the ATs which they did not perform the conduction block (90%).

**Conclusion:**

In multiple ATs, FCB regions may not be uncommon. The participation of FCB regions in the mechanism of ATs showed three different kinds of performance. The dynamic nature of this substrate may provide insight into the reasons for the high recurrence of related ATs.

## Introduction

AT related to atrial scarring and postoperative Atrial fibrillation (AF) often results in the coexistence of multiple types of AT, typically requiring more complex ablation procedures. It was previously believed that the main mechanisms for the occurrence of multiple types of AT in a patient were related to scar-associated electrically silent areas and slow conduction [1]. However, in recent years, high-resolution mapping technology has greatly improved the resolution of excitation mapping and can accurately display the excitation conduction pathway for targeted ablation. But the recurrence rate of this type of AT is still high (40–50%) [1,2]. This predicts that the occurrence and coexistence of multiple ATs may also be related to other mechanisms. In explaining the mechanisms of ventricular arrhythmias (VAs) and AFs, functional conduction block has been proven to play an important role [3]. However, there is little description of functional conduction block in AT [4,5]. In our hospital’s AT ablation cases, we prospectively conducted detailed substrate mapping and excitation mapping on cases where multiple types of AT coexist in the same patient, aiming to explore the possible mechanisms underlying the maintenance of ATs and the high recurrence rate of arrhythmias in such patients.

## Methods

### Patient characteristics

This study prospectively and continuously conducted high-density excitation mapping and substrate mapping on 105 patients with atrial fibrillation/flutter who underwent catheter radiofrequency ablation in our hospital from April 2018 to December 2021. Among these 105 patients, 13 patients (7 males and 6 females) who experienced 2 or more types of AT during the ablation process were included in this study. Cases with unstable cycle length (CL), non-sustained AT, and failed ablation were excluded. Even with the application of antiarrhythmic drugs, the AT of these patients cannot be effectively controlled. Among them, 11 patients had sustained AT, and 2 patients had paroxysmal AT, with an average duration of AT of 12 months (minimum 1 month, maximum 36 months). All patients underwent routine anticoagulation therapy before ablation and esophageal echocardiography to exclude left atrial (LA) thrombus. Oral anticoagulants were discontinued on the day of surgery, replaced with heparin during surgery, to maintain the activated partial thromboplastin time at 2–3 times the normal value. All patients provided written informed consent, and the study protocol was approved by the local Research and Human Ethics Committees.

### Electrophysiological Study and Mapping

Antiarrhythmic drugs (except amiodarone) were discontinued at least 5 half-lives before the operation; amiodarone was discontinued one month before the operation. A deflectable decapolar catheter (Dynamic XT, Boston Scientific) was positioned within the coronary sinus. Atrial mapping was performed with a Navistar Thermocool 3.5-mm D-F curve ablation catheter with Smart Touch technology (Biosense Webster) or multipolar mapping catheters (Pentaray, Biosense Webster Inc.). Bipolar electrograms filtered at 10–400 Hz were recorded. A bipolar signal from the coronary sinus (CS) electrode was used as the timing reference.

**Fig. 1:**
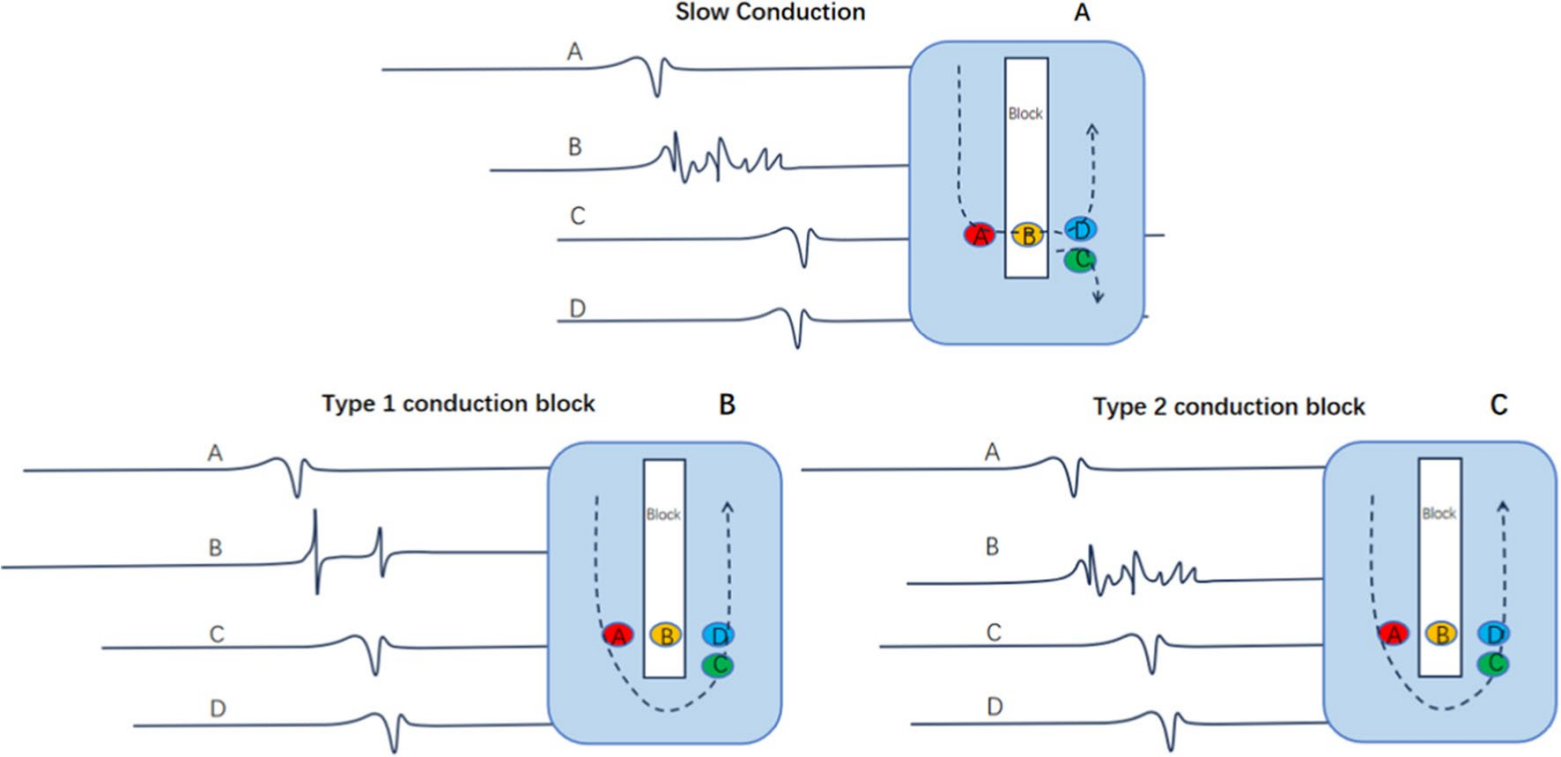
The relationship between potentials and anatomical locations in slow conduction and conduction block. A. The relationship of potentials at different positions in slow conduction. B.The relationship of potentials at different positions in the Type 1 conduction block. C. The relationship of potentials at different positions in the Type 2 conduction block

**Fig. 2:**
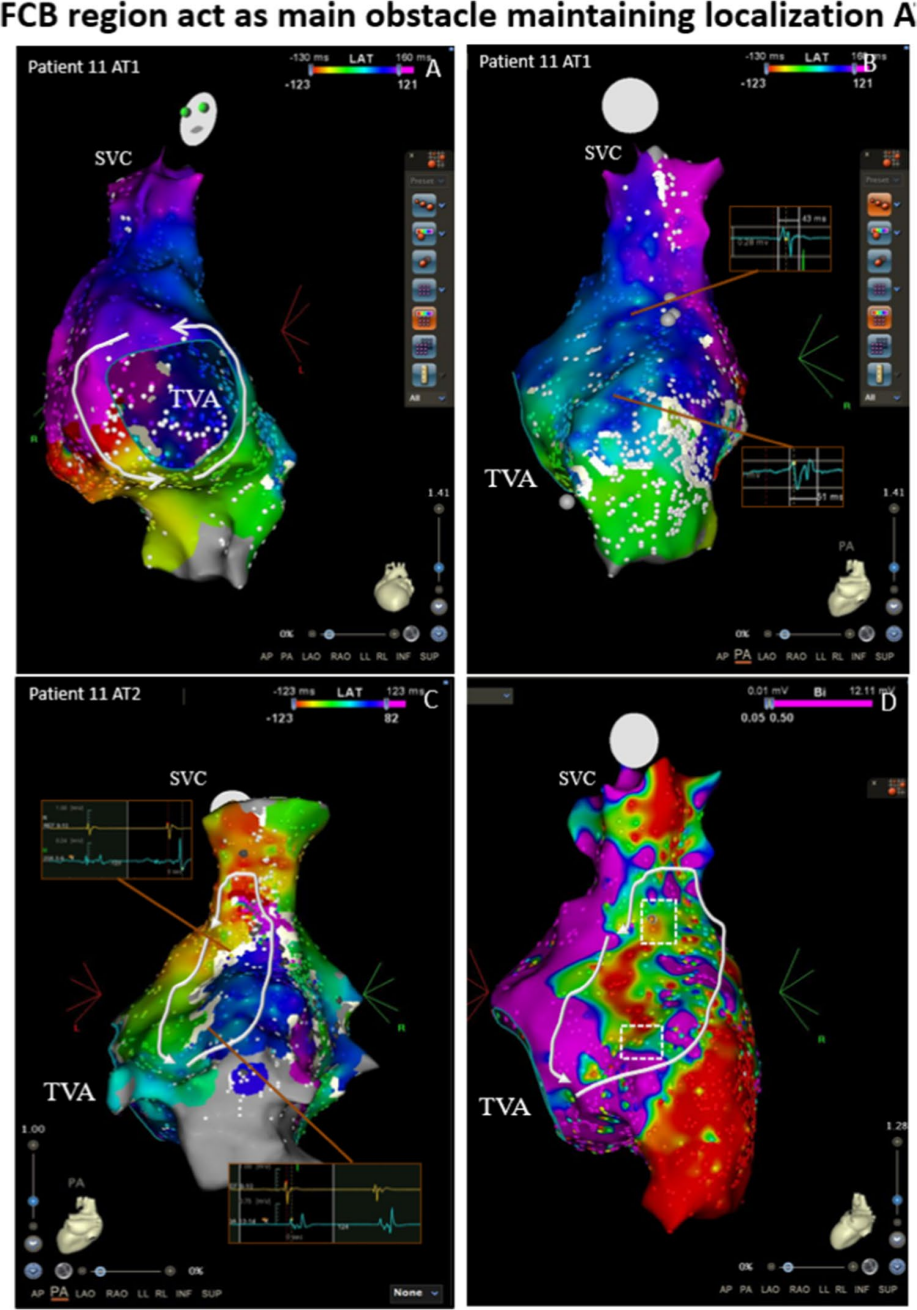
An example of the FCB region acting as the main obstacle to maintaining multiple ATs. The mapping results of two kinds of ATs in Patient 11. A, Activation map of the first AT showing a PTF. B, Activation mapping of the right atrial septum of the first AT shows that although the operative incision made conduction nonhomogeneous, forming a winding conduction from the anterior septum to the posterior septum (white arrow line), several breakouts still caused no obvious conduction block or delay. C, After ablation of the CTI, a second AT was induced through burst pacing. Activation mapping shows a localized reentry run around the obstruct line (white line), which is not shown in the first AT. The DPs are shown near the white line, while in the same location in the first AT (B), they showed low-amplitude biphasic or polyphasic potentials of short duration (potential box). Some break-out positions convert to conduction blocks that form a longer obstacle line and may more easily induce reentrant cycle rounding. This suggests that these positions may be the FCB region, which is only present in the second AT and provides an important obstacle line rounded for reentry. D, Voltage map of the second AT before ablation. The potential FCB region was related to the border zone of the scar tissue (white dotted box). AT = atrial tachycardia; CL = cycle length; CTI = cavotricuspid isthmus; DP = double potential; FCB = functional conduction block; FP = fragmented potential; PTF = peritricuspid flutter; SR = sinus rhythm; SVC = superior vena vein; TVA = tricuspid valve annulus

**Fig. 3:**
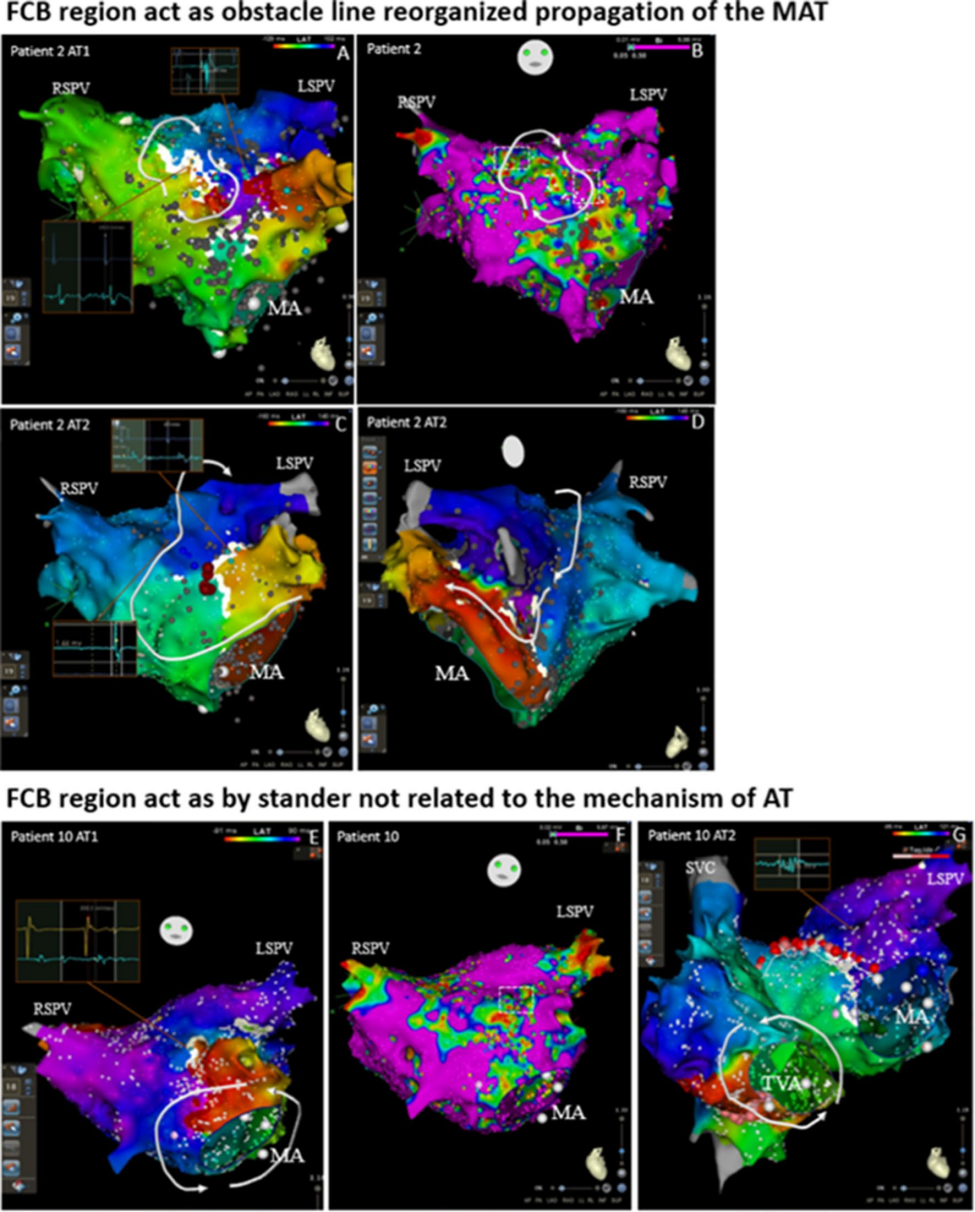
An example of FCB region acting as an obstacle line demarcating the reorganized propagation of the MAT in multiple ATs and acting as a bystander unrelated to the mechanism of AT. A-D, The mapping results of two kinds of ATs in Patient 2. A, Activation mapping showing a localized reentry run around the obstruct line formed by scars. The DPs are shown near the scar line in the first AT. B, Voltage map of the first AT before ablation. C-D, After ablation of the isthmus in the scars (marked as a red point), the AT converted to an RMAT depending on the LPV (the left atrium of these patients was scar dispersed, and there was an abundance of spontaneous scarring in the mitral isthmus and the ridge of the left pulmonary vein, which may lead to RMAT without CPVA. The location that first showed DP showed normal potential in the second AT, and the white block line disappeared due to nearby atrial re-conduction, which suggests that the upper part of the obstruct line which showed DPs in the first AT may be the FCB region, and that it may provide an important obstacle line rounded for reentry. There is a new white block line near the LAA and the location where the FP shown in AT1 converted to a DP in AT2, which suggests that this position may also be the FCB region that was only present in the second AT. Furthermore, this new obstruct line resulted in the longer conduction pathway that may make the reentrant cycle easier to maintain. After ablation, the mitral isthmus AT converted to SR. Note that there were two regions of the FCB in this patient that functioned in different mechanisms of ATs. The potential FCB region was related to the border zone of the scar tissue (white dotted box). E–G, The mapping results of two kinds of ATs in Patient 10. E, Activation mapping showing a PMF. There was an obstructed line near the LAA that showed DPs in the first AT. F, Voltage map of the first AT before ablation. G, After ablation of the mitral valve to the right superior pulmonary vein (this ablation line was chosen due to the scar at the anterior wall of the LA and the lack of electrical activity in the right superior pulmonary vein), AT converted to PTF. The location which showed DP in the first AT showed FP in the second AT, and the white block line disappeared due to nearby synchronous atrial activation, suggesting that the obstruct line, which showed DPs in the first AT, may be the FCB region and that it may not be related to the second AT. After ablation of the CTI, AT converted to SR. AT = atrial tachycardia; CL = cycle length; DP = double potential; FCB = functional conduction block; FP = fragmented potential; LAA = left atrial appendage; LPV = left pulmonary vein; MA = mitral annulus; PMF = perimitral flutter; PTF = peritricuspid flutter; RMAT = roof-dependent macroreentrant tachycardia; SR = sinus rhythm; SVC = superior vena vein; TVA = tricuspid valve annulus

**Fig. 4:**
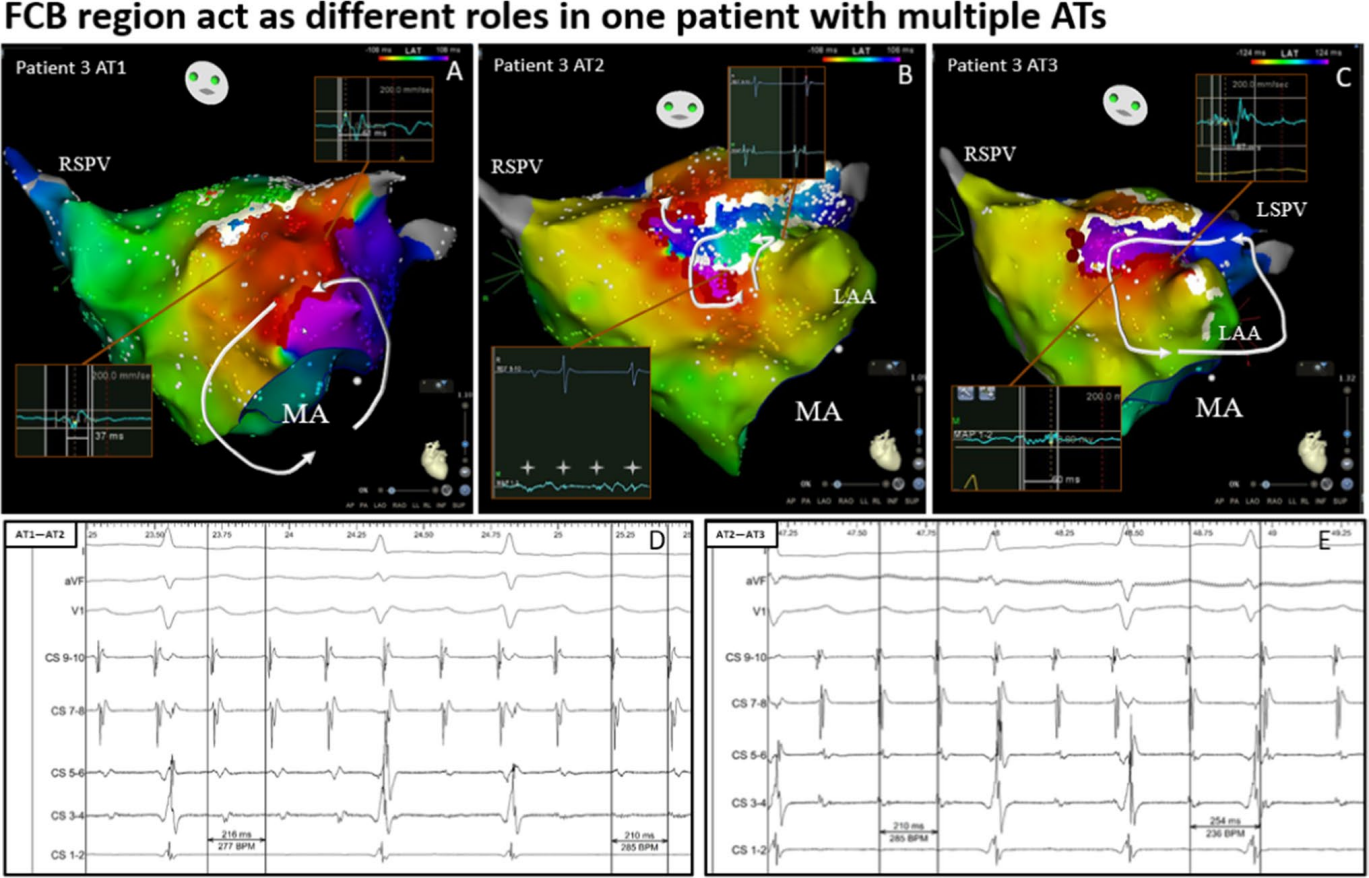
The mapping results of three kinds of ATs in Patient 3. A, Activation mapping shows that the mechanism of the first AT was PMF. B, AT 1 converted to a localized reentry with two breakouts running around the obstruct line (white line) after ablation of the mitral isthmus. A DP was observed near the white line. This suggests that this position may be the FCB region, which was only present in the second AT, and it provides an important obstacle line rounded for reentry. C, AT 2 converted to a localized reentry running around the LAA after ablation of the upper breakout, and the white block line disappeared, again causing synchronous activation near the atrium. The DP converted to the FP. Note that the disappearance of the FCB region caused the reconduction of this region, which is part of the reentrant pathway and may be one of the requirements maintaining the third AT. After ablation of the FPs near the LAA, AT converted to SR. There was a constant scar line near the LSPV (white line; the obstructing line was part of the prior incomplete roof line). D, The intracardiac electrogram showed that the first AT converted to the second AT. E, The intracardiac electrogram showed that the second AT converted to the third AT. AT = atrial tachycardia; CL = cycle length; DPs = double potentials; FCB = functional conduction block; FP = fragmented potential; LAA = left atrial appendage; PMF = perimitral flutter; PTF = peritricuspid flutter; LSPV = left superior pulmonary vein

During the reconstruction of the atrial anatomy, excitation mapping and voltage mapping were performed simultaneously, and the electrically silent area (ESA) and double potential (DP) area were specifically marked. The definition of scar in mapping is as follows: scar, < 0.05 mV; scar border zone, between 0.05 ~ 0.5 mV; and normal tissue, 0.5 mV. Double potential is defined as: two high-frequency potentials with a minimum interval of a 30 ms isopotential line. The high-frequency fragmented potential is defined as: relatively long conduction time, multi-directional reentrant bipolar potential, but the voltage is within the basic range. The slow conduction potential is defined as: relatively long duration (mostly more than 1/3 of the circumference), lower voltage, and a fragmented low-frequency waveform. However, the earliest activation potential phase locally (compared with the earliest phase of the potential in the surrounding excited area) remains the earliest phase in the fragmented potentials, indicating that local conduction is still primarily sourced from slow conduction, Fig. 1A. The conduction block is defined as: (1) Locally manifested as double potentials. The phase of the first potential of the double potentials did not occur later than the activation time on one side, and the phase of the tail potential of the double potentials did not occur earlier than the activation time on the other side, see Fig. 1B. (2) Locally manifested as fragmented potentials. Fragmented potentials generally have a longer duration, and the latest phase of fragmented potentials is significantly longer than the latest excitation phase around it (indicating complex internal conduction, the internal conduction time is too long, its output time is later than its surrounding myocardium bypass conduction time), see Fig. 1C. FCB is defined as: when subjected to excitations from different directions or different frequencies show different conduction properties. Specifically, manifested as for example, during one AT, it shows as defined conduction block, while during another AT or sinus rhythm, it does not show as defined conduction block (Figs. 2–4). In the CARTO system, lines showing as defined conduction blocks are represented by white lines. During radiofrequency ablation, if the P wave morphology or tachycardia cycle length (CL) changes, remapping is performed. Routine entrainment mapping is performed to verify the tachycardia mechanism or determine the ablation target, but it is not used when strongly suspected that entrainment will cause tachycardia change or termination.

For each area of interest, data on intracavitary bipolar electrograms (EGMs) morphology, amplitude (in millivolts), and duration (in milliseconds) were collected and analyzed. At least 5 EGMs were collected for each area of interest, and each collection area had a density of at least 15 points to verify and ensure the accuracy of the recorded data.

### Definition of the Reentry Circuit and Classification of the AT

AT is classified according to the mode of excitation into focal AT, anatomical macroreentrant AT (AMAT), and non-AMAT (localized reentry) [1]. AMAT includes cavotricuspid isthmus-dependent atrial flutter (CTI), perimitral flutter dependent on the mitral isthmus (PMF), and roof-dependent macroreentrant tachycardia (RMAT). Localized reentry is defined as: the entire tachycardia excitation cycle length can be recorded in a local area, with the size of the localized reentry circuit varying, mostly centered around scars that appear as conduction blocks [1]. Focal AT is defined as: the earliest excitation of the atrium originates from a point and spreads to the surroundings at the same time (the excitation time measured in general is less than the tachycardia cycle length) [6]. Multi-loop AT is defined as: multiple reentry loops share an isthmus, including double-loop AT and triple-loop AT, each loop excitation time meets the tachycardia circumference [6]. When a time jump phenomenon appears between the distal end of the coronary sinus and the left atrial appendage in the tachycardia excitation mapping, and this position (PPI-TCL) < 20 ms, while the left atrial anterior wall, mitral 6 o’clock-11 o’clock position (PPI-TCL) > 20 ms, is suspected to be epicardial AT involving the coronary sinus or Marshall vein [7–9].

### Catheter Ablation

Ablation strategies were adopted according to the mechanisms of ATs. Generally, the ablation of localized reentrant AT targets the isthmus that exhibits fragmented potentials (FPs) related to myocardial excitation during diastole, most of which are viable slow conduction sites left by scars or anatomical blocks. The ablation of macroreentry atrial tachycardia (MAT) selects the narrowest conductive tissue between scars or anatomical obstacles based on anatomical length, catheter stability, tissue thickness, and proximity to vulnerable structures. For focal AT, ablation is performed at the early excitation site based on mapping results. Ablation uses a saline-infused catheter with a tip length of 3.5 mm (Thermocool®ST catheter, Biosense Webster) in power mode, with an output power of 25–45 W.

Two main situations may occur during the ablation process for multiple ATs. (1) The patient is in a persistent AT attack at the start of surgery: high-density mapping is performed (to avoid termination or conversion of tachycardia, entrainment mapping is only performed when the final reentry diagnosis is confirmed). To avoid iatrogenic AT due to incomplete ablation, changes in P wave morphology, tachycardia cycle length, or reference coronary sinus excitation sequence that occur during ablation will be evaluated after completing the planned ablation. If the AT cannot be terminated or converts to a new AT after completing the planned ablation, re-mapping will be performed and mapping and ablation will be performed using the same strategy as above. If the AT terminates after planned ablation, stimulation-induced AT will be performed. The endpoint of surgery is defined as no sustainable AT can be induced by either programmed stimulation or Burst stimulation. (2) The patient was in sinus rhythm at the start of surgery: pacing-induced AT is performed. If the induced AT is clinical AT or stable sustainable AT, high-density mapping is performed and the abovementioned ablation procedure is executed.

### Postoperative management and follow-up

Patients underwent continuous electrocardiographic monitoring during their postoperative hospital stay until discharge, then continued treatment with the anticoagulant and rhythm control drugs they were already taking preoperatively. Three months after discharge, if there was no recurrence of arrhythmia or symptoms related to arrhythmia, the use of rhythm control drugs will be discontinued. In addition, if conditions permit, anticoagulant therapy with warfarin or dabigatran etexilate will also be discontinued. All patients received follow-up examinations in the outpatient clinic at 3, 6, and 12 months after ablation. At each follow-up, patients underwent 24-h Holter monitoring and echocardiography. In cases where symptoms suggestive of an arrhythmia attack occur, additional 12-lead electrocardiogram (ECG) examinations or multiple Holter monitorings are performed to confirm the recurrence of AT.

### Statistical analysis

All continuous variables are expressed as mean ± standard deviation or median. Statistical differences were evaluated using an ANOVA. P < 0.05 was considered statistically significant. Statistical testing was performed with SAS version 9.2.

## Results

### Patient characteristics

The clinical characteristics of the 13 patients included in the study are shown in Table 1. Multiple reentrant circuits occurred in patients with a history of postoperative disease (8 patients) and those without a history of previous surgery or catheter intervention (5 patients). One patient had dilated cardiomyopathy with left bundle branch block (LBBB) and received CRT-D treatment. One patient underwent surgery due to atrial myxoma. One patient underwent cavotricuspid isthmus (CTI) ablation surgery due to typical AT. Six patients underwent AF ablation, and another five patients had no obvious structural heart disease on echocardiography and had no history of previous surgery or catheter intervention. Six patients underwent AF ablation without any ablation based on fragment electrogram.

**Table 1:** Clinical characteristics

Patient	age	sex	Heart disease	Left atrial size, mm	EF, %	History of previous ablation
1	78	Male	DCM, LBBB	38.1	37	None
2	37	Male	None	38.6	52.4	None
3	53	Male	AF, HF, SN	45.4	35	CPVI, Roof line
4	54	Female	AF	45	42	CPVI
5	67	Female	AF	49	38	CPVI
6	38	Male	AF	38.9	62	CPVI
7	64	Male	None	53.1	30.4	CTI
8	59	Female	AF	48	45	CPVI
9	56	Male	None	44.9	66	None
10	52	Male	None	47.5	61	None
11	64	Female	After surgery	37.5	64	None
12	58	Male	AF	42	59	CPVI, Roof line
13	35	Female	None	36.6	43	None

AF = atrial fibrillation; CPVI = circumferential pulmonary vein isolation; HF = heart failure; LBBB = left bundle branch block; SN = sinus node disease; CTI = cavotricuspid isthmus

### Mapping Results

The mapping results of AT are shown in Table 2. Among these 13 patients, a total of 33 types of persistent AT could be mapped. During ablation, 7 patients presented with 2 different types of AT, 5 patients presented with 3 different types, and 1 patient presented with 4 different types. Among these patients, 17 had new ATs that transformed after ablation, and 3 had new-onset ATs induced after ablation. In the first mapping, 5 cases had localized ATs related to scars, 5 cases were MAT related to the mitral annulus, 2 patients presented with MAT dependent on the tricuspid annulus, and 1 case presented with RMAT dependent on the left atrium. In the second type of AT, 5 cases were localized ATs related to scars, 6 cases were MAT, and 2 cases were Focal AT. There were 6 cases with a third type of AT, among which 2 cases were localized ATs related to scars, 2 cases were RMAT, and 2 cases were focal ATs. One patient had four types of AT, which manifested as localized AT related to scars.

**Table 2:** AT mapping results

Patient	Number of AT	Type of AT	Cycle Length, ms	Appearance	FCB location	Ablation site	Scar area	Result
**1**	2	1 Localized	190	persistent	None	Linear ablation (ESA– RPV)	Anterior wall + Atrium roof	SR
		2 Localized	265	converted	Near the roof line	FP (Anterior wall scar)		
**2**	2	1 Localized	220	persistent	Anterior wall (near atrium roof)	FP (Anterior wall)	Anterior wall + Mitral isthmus	SR
		2 RMAT	300	converted	Anterior wall(near left atrial appendage)	FP (Mitral isthmus)		
**3**	3	1 PMF	215	persistent	None	Mitral isthmus	Anterior wall + Atrium roof	PM
		2 Localized	210	converted	Anterior wall(near LAA)	FP(near atrial roof)		
		3 Localized	254	converted	None	Near LAA		
**4**	2	1 Localized	230	persistent	None	FP (Anterior wall scar)	Anterior wall	SR
		2 Localized	260	converted		FP (Anterior wall scar)		
**5**	2	1 PTF	280	induced	None	CTI line	Anterior wall	SR
		2 Focal	280	converted		Anterior wall scar		
**6**	3	1 PMF	190	persistent	None	Mitral isthmus	Atrial roof	SR
		2 RMAT	190	converted		atrium roof		
		3 RMAT	220	converted		FP (Atrial roof scar)		
**7**	4	1 PMF	220	induced	None	Mitral isthmus	Anterior wall	SR
		2 PMF	220	converted	Anterior wall	Anterior wall line		
		3 RMAT	220	converted	None	Atrium roof line		
		4 Localized	220	converted	None	Near LAA		
**8**	3	1 PTF	210	persistent	None	CTI line	None	SR
		2 PMF	220	converted		Mitral isthmus		
		3 Focal	250	converted		LIPV Near the posterior wall		
**9**	3	1 Localized	310	persistent	None	FP (Anterior)	Anterior wall	SR
		2 Focal	250	induced	None	Anterior wall scar		
		3 Focal	210	converted	Atrial roof ( near RSPV)	Anterior wall scar		
**10**	2	1 PMF	180	persistent	Anterior wall(near LAA)	Anterior line; RPV	Anterior wall	SR
		1 PTF	190	converted	None	CTI line		
**11**	2	1 PTF	280	persistent	None	CTI line	Atrial septum	SR
		2 Localized	240	induced	Right atrial septum	FP (atrial septum)		
**12**	2	1 RMAT	210	persistent	None	Roof line	Atrial roof	SR
		2 PTF	245	induced		CTI line		
**13**	2	1 Localized	300	persistent	None	FP (atrial roof scar)	None	SR
		2 Localized	250	converted		FP (Anterior wall scar)		
		3 PTF	245	converted		CTI line		

MAT = macroreentry atrial tachycardia; CTI = cavotricuspid isthmus; DP = double potential; ESA = electrically silent area; FCB = functional conduction block; FP = fragmented potential; LAA = left atrial appendage; PM = pace marker; PMF = perimitral flutter; PTF = peritricuspid flutter; RPV = right pulmonary vein; RMAT = roof-dependent macroreentrant tachycardia; SR = sinus rhythm

### Occurrence and Manifestation of FCB in Multiple ATs

In this series of cases of multiple ATs, some areas showed complete conduction block only during specific AT attacks, and showed continuous conduction or slow conduction but not complete block in sinus rhythm or other ATs, suggesting a manifestation of FCB. Among the 13 patients, 7 (54%) showed FCB during AT conversion. Most of these FCB areas seemed to be near scar areas (spontaneous or iatrogenic). According to the excitation mapping results, the mechanism of FCB areas involved in maintaining AT can be summarized into three situations. Firstly, the FCB area serves as the main obstacle for maintaining the circular reentry pathway (as shown in Fig. 2), and this phenomenon occurred in 3 out of 7 patients.

Second, the FCB area plays the role of an obstacle line in the reentry pathway, significantly prolonging the total reentry path (as shown in Fig. 3A-D). This phenomenon occurred in 6 out of 7 patients. This transformation of the reentry pathway caused by the appearance or disappearance of the FCB area may participate in anatomical macroreentrance, as shown in Fig. 3.

Thirdly, the FCB area acts as a bystander and does not affect the main reentry circuit path, which may be unrelated to the mechanism of AT(as shown in Fig. 3E-G). This phenomenon occurred in 2 out of 7 patients.

The FCB area may have different functions in different types of AT in the same patient (as shown in Fig. 4).

The incidence of FCB phenomena and the role of FCB areas shown by mapping in different cases are summarized in Fig. 6.

An analysis of the potential FCB areas during AT and sinus rhythm that did not manifest as blockages is shown in Table 3. 10% of potential FCB areas EGM showed near-normal voltage, amplitude 0.692 ± 0.382 mV, duration 31.833 ± 11.035 ms; a total of 53.3% of EGM showed low voltage, amplitude 0.241 ± 0.107 mV, duration 41.813 ± 10.431 ms. 20% of EGM showed complex fragmented electrical conduction, presenting multiple rapid peaks, amplitude 0.523 ± 0.317 mV, duration 66.750 ± 15.316 ms. 10% of EGM showed slow electrical conduction, presenting low-amplitude long-range fragmented potentials, amplitude 0.103 ± 0.058 mV, duration 75.9 ± 15.007 ms.

**Table 3:**
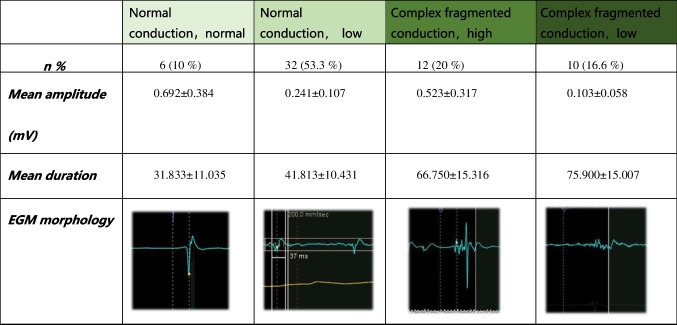
Voltage characteristics of the potential FCB areas

### Catheter Radiofrequency Ablation

The ablation strategy depends on the results of the excitation mapping. The local reentrant AT ablation mainly seeks the residual isthmus, which is mostly located between two anatomical obstacles, such as natural valve rings, pulmonary veins, or between the ESAs area, often showing fragmented potentials. In ablation related to large reentry, the shortest or easiest ablation line is typically designed for catheter operation, forming a barrier between anatomical structures or scars. In five cases of mitral valve-related AT ablation, there were ablation lines connecting the mitral valve to the left superior pulmonary vein (n = 4) and the mitral valve to the right superior pulmonary vein (n = 1). In the ablation of roof-related reentry, the ablation lines connect the upper pulmonary veins on both sides of the left atrial roof (n = 4), and the upper pulmonary vein to the mitral valve annulus (n = 1). Focal AT was ablated at the earliest excitation site (n = 4). During mapping, the FCB area is often not the target ablation area, but the target area for ablation may be residual conduction tissue near the FCB area (Fig. 3). None of patients underwent substrate modification ablation during sinus rhythm or AT onset.

### Follow-up

There were no procedure-related complications. All 13 patients were in sinus rhythm after ablation. After programmed stimulation and burst stimulation induction, 11 of these patients were noninducible; atypical nonsustained flutter was inducible in 1 patient, and nonsustained AF was inducible in 1 patient. A pacemaker was implanted in 1 patient due to sinus bradycardia 3 days after ablation. With a mean follow-up of 12 months, 9 patients were successfully treated without the use of antiarrhythmic drugs. ATs reoccurred in 4 patients (31%).

## Discussion

### Main findings

In a series of cases with multiple ATs, we observed that 7 patients (54%) exhibited the phenomenon of existing FCB regions. This may suggest that in cases of multiple ATs, especially in scar-related AT, FCB may be a not uncommon electrophysiological phenomenon. Among the 18 cases of AT that occurred in these 7 patients, the mechanisms of 11 ATs (61%) were related to the conversion of the FCB region (Fig. 6). According to the results of excitation mapping, the FCB region can play three different roles in the mechanism of AT. First, the FCB region serves as the main central block area for maintaining local reentry, which is obviously directly related to the maintenance of AT (27%). Second, the appearance of the FCB region may serve as a barrier line, reorganizing the conduction pathway of local AT or large reentry AT. This may result in the potential taking more time to conduct to the native location prolong the reentry time (Figs. 3C and D), making reentry easier to maintain or more likely to occur (55%). Third, the FCB region acts as a bystander and may not be significantly related to the maintenance of AT (18%). The FCB is a manifestation of anisotropic conduction in local tissues, and this anisotropic conduction is often related to different directions of excitation sources and different input frequencies. In multiple ATs, each type of AT may have different cycle length and conduction path, which makes this anisotropic manifestation more likely to occur, greatly increasing the probability of the appearance of FCB regions. In our research results, potential FCB regions may be involved in mediating 84% of the maintenance of multiple ATs, and this special mechanism may be one of the mechanisms by which a single patient has multiple ATs. Currently, most ablation strategies are still mainly based on “what we can see”, relying more on the results of excitation mapping at the time of AT. The special performance of the FCB region only appears under specific conduction modes or frequencies. This unpredictability may be ignored in actual operation procedures and may be one of the reasons for the high recurrence rates related AT.

### Occurrence of the FCB and Substrate Characteristics

**Fig. 5:**
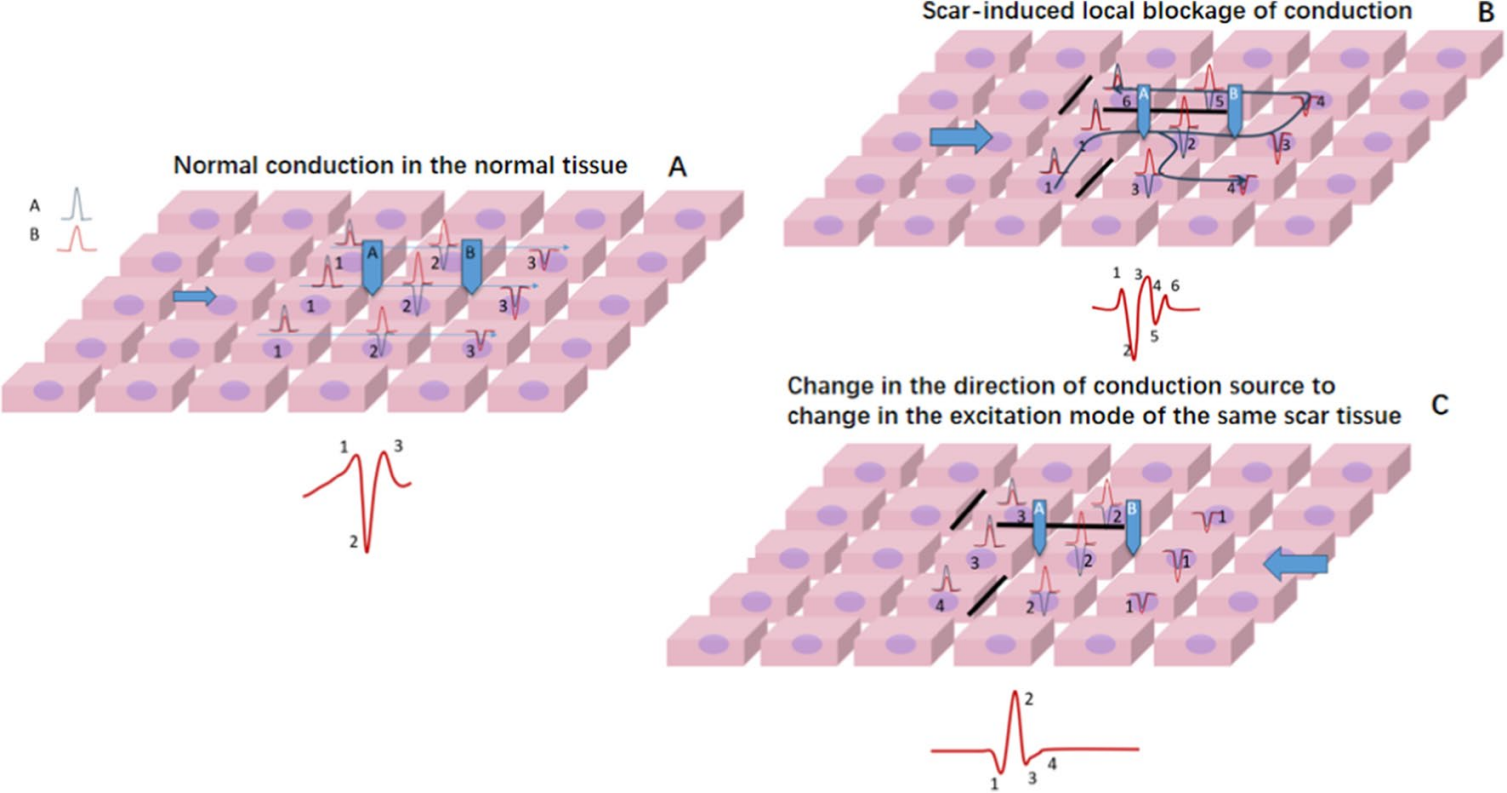
The manifestation of anisotropic conduction between different tissues with different conduction directions. A, In the conduction process of normal tissue, the excitation from the left simultaneously excites the subsequent row of cells. The entire excitation process occurs within 3 units of time, forming a narrow potential normal conduction potential. B, In the tissue where the scar causes local block (black line), the excitation from the left, due to the local block, needs 6 units of time to complete the excitation of the entire piece of cells. The voltage is discretely distributed over time, forming fragmented potentials. C, In the same tissue where the scar causes local block (black line), the excitation from the right can complete the excitation of the entire piece of cells within 4 units of time. The potential is similar to normal potential. This shows that different excitation source directions can produce conduction heterogeneity. Note: This scheme demonstrated intracellular conduction dispersion, with scar isolation being just one of the reasons for this dispersion. The scheme also includes frequency dependence, which can cause local cell conduction interruption. Frequency-dependent conduction interruption can be a disease of the cell itself (cannot accept a higher frequency response), or it can be scar-induced (a local scar causes intracellular discrete conduction, which delays the outgoing time significantly, forming a local 8 reentry)

It is not difficult to observe that in multifocal AT, the scar substrate often participates in the conduction process. Scar tissue can increase the anisotropy of tissue conduction, and the local tissue conduction anisotropy manifests as: when receiving excitation from a certain direction or different input frequencies, it may include 1. Local conduction time is relatively prolonged; 2. An increase in the voltage dispersion; 3. The amplitude of the voltage decreases per unit time. During mapping, the characteristics of bipolar potentials will be displayed as changing from simple less-amplitude inflection potentials to complex multi-amplitude fragmented potentials (Fig. 5). These local characteristics may cause prolongation of local conduction time or further block. In the analysis of potentials in potential FCB areas (Table 3), 90% of the locations showed low voltage or complex potentials. A total of 53.3% of the areas show low voltage, which may be caused by local scars. When low voltage manifests as simple potentials (few positive and negative deflections) and is accompanied by a short potential duration, it suggests that severe scarring can lead to a reduction in the number of excitable cardiomyocytes, resulting in low voltage. When anisotropy occurs at this location, it is more likely to cause source-sink mismatch, thereby more easily to form local block. 20% of the areas show high-frequency complex potentials, which are mainly produced due to complex conduction formed by the intersection between different layers of myocardium. Its potential duration is slightly longer, mainly due to more (thicker) local myocardium. Such locations are also more likely to have anisotropic conduction, but the conditions for forming local block may be stricter. 10% of the areas show low-frequency, long-range complex potentials, which suggests that anisotropic delay conduction has occurred in the current state. When changing conduction conditions (such as the excitation direction), it may convert to near-normal conduction (Fig. 5C) or form a local block.

**Fig. 6:**
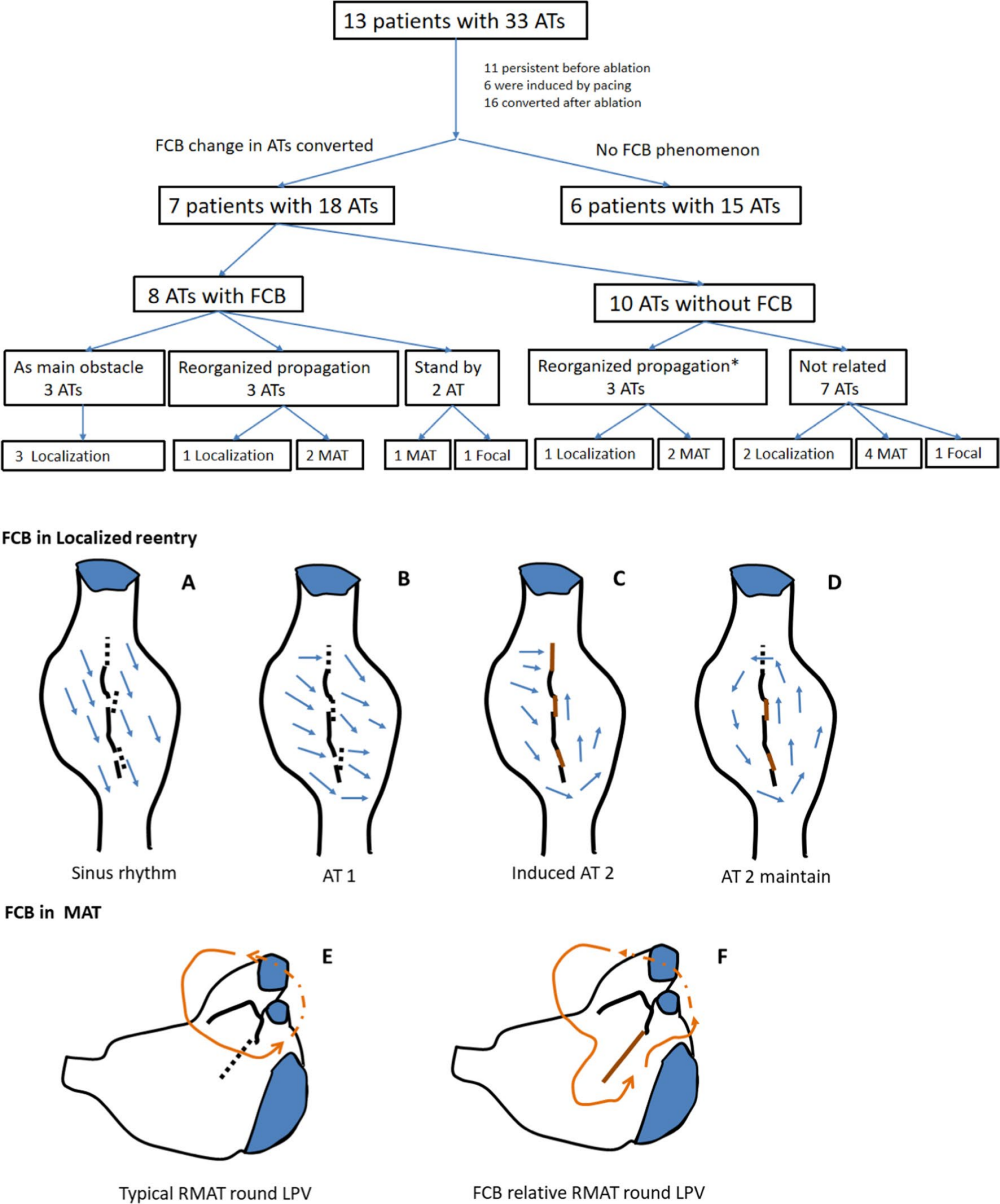
Mapping results and the roles of FCB regions acting in different cases, and the schematic of the FCB acting in ATs. * The appearance or disappearance of both may reorganize the conduction pathway of the reentrant cycle. In 10 ATs without FCB, 3 ATs, including 2 MATs and 1 localization AT, may be sustained due to the reconduction of the FCB regions. A, Conduction pattern near the scar of the right atrial septum during sinus rhythm. The time to reach the tissue of the two sides of the scar was the same (the underlying FCB regions are marked with black dotted lines). B, The conduction pattern near the scar of the right atrial septum during AT 1 was not related to scarring. During AT 1, the conduction direction changed according to the different times required to reach the tissue of the two sides of the scar; at this time, the underlying FCB region could still conduct with no obvious time interval difference. C, During induced pacing or after ablation, the conduction first blocked the isthmus and underlying FCB regions (the upper brown line) and made the conduction pathway downward; at this time, underlying FCB regions showed conduction blocks. D, Due to the sustained conduction block of the FCB regions and the long pathway that allowed the isthmus to recover conduction after CL, localized reentry could be sustained. E, Typical RMAT with a round LPV; the conduction pathway is relatively short. F, Once the underlying FCB region showed conduction block, the reentrant pathway length may have made the reentry easier to induce or sustain. AT = atrial tachycardia; CL = cycle length; MAT = macroreentry atrial tachycardia; FCB = functional conduction block; LPV = left pulmonary vein; RMAT = roof-dependent macroreentrant tachycardia

FCB regions are often found near scar areas or near the intersection of different anatomical structures (such as near the base of the atrial appendage). The main role of FCB in local reentry is to provide continuous (Fig. 1) or extended (Fig. 2) block lines, allowing the tissue to recover conduction within the propagation time provided by the obstacle, thereby satisfying the minimum cycle length of reentry (Fig. 6A). In macroreentrant AT, the block area provided by the FCB will change the conduction path, generally prolonging the conduction time, which may make it easier to maintain the macroreentrant circuit (Fig. 6B).

The scar may be caused by myocardial injury or by iatrogenic surgery. Some new scars produced by intraoperative ablations can lead to the occurrence of multiple AT. For example, the 8th patient, who had 3 ATs, the first of which was tricuspid valve-dependent AT, underwent tricuspid isthmus ablation. It then transformed into mitral flutter, which is obviously unrelated to tricuspid ablation. After the subsequent isolation of the mitral isthmus, it transforms into a focal AT of the lower left pulmonary vein, which may be related to the scar caused by the mitral isthmus line.

We can predictively describe the different manifestations of FCB phenomena in multiple ATs, mainly because the alternation of various ATs provides opportunities to show different conduction characteristics of potential FCB regions under different circumstances. In fact, in addition to mediating the conversion between different ATs, FCB is also the reason for the occurrence of AT that alternate with changes in cycle length [10–12].

### Ablation of Multiple ATs

Multiple ATs are often associated with more than two reentry circuits, which often involves more complex ablation procedures^1^. Our ablation strategy in the study still relies on the results of excitation mapping. Despite high-density mapping, only one patient (double-loop local reentry, FCB region not shown) could predict the reentry circuit of the second AT before the first ablation. The instability of the functional area makes the second or third AT unpredictable. The main role of the FCB region is to maintain the path of reentry. After mapping, these areas are usually not the target sites for ablation. Meanwhile, if substrate ablation is performed in these areas, the current AT may not be terminated. The ablation site still depends on the narrowest conduction tissue between the reentry isolation line or scar or anatomical obstacles. In the context of stable atrial tachycardia, the FCB can manifest, which essentially represents the anisotropic conduction of this area (Fig. 5). In potential morphology, it exhibits fragmented potentials under conduction in a certain direction. We hypothesize that this may be based on the same principle as the varying potentials exhibited at the same location during atrial fibrillation at different times. Atrial fibrillation, compared to multifocal atrial tachycardia, involves a greater number of excitation directions (and frequencies), which can maximally reveal the anisotropy of the local tissue. In some cases of persistent atrial fibrillation, it is common to observe that at some locations, the potential morphology does not significantly change during the fibrillation process. However, other locations intermittently exhibit fragmented potentials of prolonged duration, suggesting significant anisotropy of the substrate at these sites. These locations may indicate targets for substrate modification ablation. Such ablation choices are reasonable, because the prolonged discrete conduction within the tissue at these sites could potentially form the basis for local micro-reentry. In multiple types of atrial tachycardia, the FCB merely mediates local block (which may manifest as prolonged fragmented potentials or double potentials), and the actual ablation targets are inevitably the isthmus areas adjacent to the block. However, this does not imply that the FCB areas are not worthy of ablation. They still possess characteristics that could potentially lead to local micro-reentry. Future studies will require higher resolution mapping to solve these critical questions, such as identifying which types of complex fragmented potential are essential for maintaining arrhythmia and which types do not influence the occurrence of tachycardia, regardless of changes in conditions (e.g., in cases of atrial fibrillation or atrial tachycardia with extensive scarring, where complex fragmented potential is abundant).

In cases with multiple atrial tachycardia of this study, FCB is a special manifestation of the substrate, and its instability may lead to an unpredictable AT, which may be a reason for the relatively high recurrence rate of multiple ATs. In addition, the occurrence of FCBs is related to the direction of excitation and frequency. Entrainment mapping may cause AT to convert, making the mechanism of clinical AT complex and not recommended for use. Judging the FCB region by substrate mapping under sinus rhythm or artificially different cycle length pacing may have lower effectiveness because individual FCB regions only work for their matching AT. Therefore, providing more conduction conditions with different pacing strategies to induce possible ATs and performing targeted ablation may be more effective in reducing the recurrence rate than performing substrate ablation under sinus rhythm. Future computer simulations may be used to identify the conditions and intervention strategies required for FCB.

## Limitation

This study has several limitations: (1) It only contain the patient group who underwent radiofrequency catheter ablation, and the number of patients was limited. (2) Only cases that were successfully ablated from the original pool were included. While this strategy provided more confidence in the proposed mechanism for patients who were successfully ablated, it also indicates that in several patients, this strategy might not be fruitful if not mechanistically accurate. Cases that fail to achieve successful ablation after repeated mapping may involve other overlooked factors contributing to tachycardia, such as epicardial structures or limitations of the current mapping resolution. In this study, direct epicardial mapping was not performed on patients suspected of having extracardiac connections. (3) This study analyzed the conduction substrate of multiple ATs. Although we noticed that the incidence of FCB regions in multiple ATs is relatively high (54% in our study), we did not systematically evaluate the FCB phenomenon before ablation. In addition, there is a lack of effective measures for identifying FCB without specific AT attacks. (4) Although the FCB is related to atrial scars, it is not the target ablation site under excitation mapping. However, as a strategy, ablation targeting the FCB region to avoid recurrence of other ATs still requires careful consideration. (5) In this study, most mapping results were performed with Penta-Ray catheter, and the definition of most scar area were < 0.05 mV. Although in some cases this definition was relaxed to a voltage of 0.02 mV due to controversial positions with low-amplitude signals, this may filter out some important information, leading to a different activation map. Using smaller electrodes may improve the resolution.

## Conclusion

In multiple ATs, the FCB phenomenon may be a common electrophysiological feature, especially for scar-related ATs. According to the results of excitation mapping, FCB regions can describe three manifestations in the maintenance mechanism of multiple ATs. First, the FCB region acts as a central block, maintaining the reentry circuit. Second, the FCB region acts as a barrier line, which may reorganize the propagation of reentry. Third, the FCB region acts as a bystander and may be unrelated to the mechanism of AT. FCB is a manifestation of local block conduction anisotropy, and this dynamic property of the substrate may be one of the most important reasons for the high recurrence rate of related ATs.

## Data Availability

The datasets used and/or analysed during the current study are available from the publisher on reasonable request.
